# Under adverse conditions, older small tree finch males (*Camarhynchus parvulus*) produce more offspring than younger males

**DOI:** 10.1111/eth.13069

**Published:** 2020-07-17

**Authors:** Christian Wappl, Arno Cimadom, Nikolaus Filek, Eileen Heyer, Sabine Tebbich

**Affiliations:** ^1^ Department of Behavioural Biology University of Vienna Vienna Austria

**Keywords:** breeding success, Darwin's finches, invasive species, male age, parasitism, predation

## Abstract

Females of many bird species prefer mating with older males, presumably because they provide superior parental care and possibly superior genes. A previous study found that female small tree finches (*Camarhynchus parvulus*) preferred pairing with old males and had a higher breeding success when paired with old males because their nests were more concealed, higher up in the canopy and therefore less likely to be depredated. However, causes for brood loss have changed over the last decade: predation of small tree finch nests has decreased, whereas brood losses due to parasitism by the invasive parasitic fly *Philornis downsi* have increased. In the present study, we investigated (a) how the change in predation and parasitism by *P. downsi* influenced the breeding success of small tree finches, (b) whether there were still differences in breeding success between young and old males, (c) whether *P. downsi* infestation had a differential effect on nests of young and old males and (d) whether young and old males differed in foraging success. During 2012–2016, we found an overall low influence of predation and a high influence of *P. downsi*, but neither differed between nests of young and old males. Nests of old males had more fledglings than those of young males. However, the difference in breeding success disappeared when *P. downsi* numbers were experimentally reduced by injecting an insecticide into nests. This indicates that older males were able to compensate for the detrimental effects of parasitism.

## Graphical abstract

In many bird species, females paired with older males have a higher breeding success. Small tree finches are heavily affected by an invasive nest parasite. Nests of older males had more fledglings than those of younger males. However, the difference in breeding success disappeared when parasites were experimentally reduced with the insecticide permethrin. This indicates that older males are better at compensating for the detrimental effects of parasitism and shows that older males are more successful only under adverse conditions.

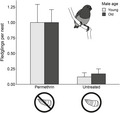

## 1 INTRODUCTION

Reproductive performance in birds increases with age during the first years of reproduction, before reaching a plateau and ultimately, in some species, declining (Forslund & Pärt, 1995). In many species, females prefer mating with older males (Hansen & Price, 1995). This may give them access to certain benefits, such as superior parental care (Gil, Cobb, & Slater, 2001) and superior genes (Manning, 1985), although the latter has been disputed (Brooks & Kemp, 2001). Females base their choices on age‐dependent signals. These signals can be acoustic, such as song rate and amplitude in the rock sparrow, *Petronia petronia* (Nemeth, Kempenaers, Matessi, & Brumm, 2012), or visual, such as the brightness of UV‐blue ornaments in eastern bluebirds, *Sialia sialis* (Siefferman, Hill, & Dobson, 2005). In small tree finch (*Camarhynchus parvulus*) males (but not females), the amount of black plumage on chin and crown increases annually (Kleindorfer, 2007; Langton & Kleindorfer, 2019). Kleindorfer (2007) found that female small tree finches have a preference for black (and therefore older) males and a higher fledging success when paired with black males in a pooled data set of the years 2000, 2001, 2002 and 2004. The higher fledging success was the result of reduced predation at black males’ nests, which were more concealed and positioned higher up in the canopy. Kleindorfer (2007) also found that females paired with brown males had larger clutch sizes and argued, among other things, that nest size determines clutch size and brown males build larger nests.

However, environmental factors have changed since 2004. A study by Cimadom et al. (2014) revealed that predation was no longer a major cause for nesting failure in the small tree finch in 2010 and 2012. Instead, the parasitic fly *Philornis downsi* was responsible for 56% of dead chicks and effectively replaced predation as the main cause of nesting failure (Cimadom et al., 2014). This invasive fly species is considered one of the most pressing problems for the avifauna of the Galápagos archipelago (Causton et al., 2006). *Philornis downsi* lays its eggs in birds’ nests, where its larvae feed upon the blood and tissue of the nestlings, leading to lower haemoglobin levels (Dudaniec, Kleindorfer, & Fessl, 2006; Fessl, Kleindorfer, & Tebbich, 2006; Koop, Huber, Laverty, & Clayton, 2011), malformations of beak and naris (Galligan & Kleindorfer, 2009) and total brood loss (Dudaniec et al., 2006; Fessl et al., 2006; Kleindorfer & Dudaniec, 2016). In addition to complete nesting failure, a large percentage of nests suffer partial brood loss due to infestation with *P. downsi* (Cimadom et al., 2014). Kleindorfer (2007) did not investigate parasitism by *P. downsi* in detail, but found a higher rate of nesting failure with dead nestlings in nests of brown (and therefore younger) males. This can probably be attributed to *P. downsi* parasitism and suggests that nests of brown males were either infested with more *P. downsi* larvae or that black males could somehow compensate for the negative effects of the parasite.

In the light of increased parasitism and decreased predation pressure, we aimed to investigate whether older males still have higher breeding success than young males and an advantage when confronted with *P. downsi* parasitism. We hypothesized that old small tree finch males are better at compensating for the negative effects of *P. downsi* and, thus, are more successful than young males in the presence of parasites.

Increasing parental food provisioning is one possibility to compensate for the high energy loss caused by *P. downsi* parasitism (Knutie et al., 2016). Parents could enhance food provisioning through higher foraging success. Age‐specific improvement in foraging success is well documented in birds (Wunderle, 1991). Females could attempt to compensate for the effects of *P. downsi* by adjusting clutch size. When nestlings are subject to high parasite pressure, females should lay larger clutch sizes to dilute the effect of the parasites on the individual chicks (Richner & Heeb, 1995). However, Forbes (1993) hypothesized that parasitized birds should shift investment towards parasite defence and survival, which could cause females to lay smaller clutches (Moss & Camin, 1970). It is also possible that, in the absence of strong predation pressure, females increase clutch size when paired with a preferred higher‐quality (and in this case older) mate (Andersson, 1994).

In the current study, we specifically tested (a) whether nests of old small tree finch males still had a higher breeding success than those of young males, (b) whether old males have higher foraging efficiency than young males and (c) whether there was a differential influence of *P. downsi* on the breeding success of young and old males. We addressed the latter question with an experimental approach by eliminating *P. downsi* larvae from 40 nests. We predicted (a) higher breeding success of older males in parasitized nests and (b) that this difference in breeding success decreases when the parasite load is reduced experimentally. We also tested whether the changes in conditions have led to a change in clutch size and whether clutch size of females paired with younger and older males differed. However, we were not able to make a directed prediction due to the diverging theories.

## 2 METHODS

### 2.1 Study site and study species

The study was conducted in the humid highlands of Santa Cruz Island, Galápagos, near Los Gemelos (0°37’34”S, 90°23’10”W; elevation approx. 500–600 m a.s.l.) in an area of approximately 20 ha. The study site is part of the *Scalesia* zone, named after the dominant tree species *Scalesia pedunculata* (Asteraceae). Data were collected during the breeding season in 2012 (January–March), 2014 (January–April), 2015 (January–May), 2016 (January–April) and 2017 (January–April).

In small tree finch males, the amount of black plumage on chin and crown increases annually (Langton & Kleindorfer, 2019), forming six distinct age categories which can be visually distinguished in the field (Kleindorfer, 2007). Kleindorfer (2007) found that 18 out of 24 individuals recaptured in consecutive years had increased the amount of black plumage exactly by one category, while two individuals had increased it by two categories and four had not increased it at all. Therefore, the age of male birds can be somewhat reliably determined in the field without capturing and/or tracking the same individual over multiple years, making the species ideal for studying the effects of male age. Using a capture–recapture method, Langton and Kleindorfer (2019) found a minimum longevity of 15 years for male small tree finches. The maximum number of years between recaptures was 7 years for females; however, their minimum longevity is unknown as age of females at first capture could not be determined. Due to their longevity, it is probable that male small tree finches remain reproductively active over a prolonged period of time.

Small tree finch males build a display nest, next to which they will sing in order to attract females (Christensen & Kleindorfer, 2007). After pair formation, the pair will build a new nest at a new location together, with the male constructing much of the coarse outer structure and the female forming the soft interior of the nest. Rarely, the pair will continue using the display nest (pers. observation). Assortative mating regarding morphological traits (bill and tarsus length) is known to occur in this species (Christensen & Kleindorfer, 2007), but no data exist on age‐dependent assortative mating. Males play an important part in raising the offspring, as they provide food both for the chicks and the female during this time (Heyer, Cimadom, Wappl, & Tebbich, 2020).

### 2.2 Nest monitoring

The area was searched for active nests of the small tree finch. Nest monitoring followed the exact method described in Cimadom et al. (2014). Nest height was estimated in intervals of one metre. Once a nest was confirmed as incubating or feeding, a pole‐mounted endoscopic camera (dnt Findoo 3.6) was used to assess the number of eggs or nestlings and whether the nestlings were alive. For nests which were found with nestlings, clutch size was inferred from the number of chicks, as hatching success was high, ranging between 81% and 97% over all four study years. Age of chicks was determined by the age of oldest chick at the time breeding activity terminated at a given nest. For nests where hatching date was unknown, chick age in days was estimated based on images of nestlings and dead chicks of known age, following the method outlined by Cimadom et al. (2014). Nests were monitored until breeding activity ended due to fledging of the chicks or failure. To optimize information and minimize disturbance, monitoring intervals were adjusted to the status of the nest. The intervals were five days for nests in the process of construction, three days for nests during incubation, two days for nests with chicks and daily when close to fledging. Nesting outcomes were classified in the same way as Kleindorfer (2007): fledged (nest empty and chicks ≥8 days), abandoned (nest and eggs intact, no parental activity), dead (nest intact and dead chicks inside the nest) or depredated (nest destroyed and/or chicks ≤7 days of age missing from the nest). After breeding activity had stopped, nests were cut down, collected in separate plastic bags and subsequently dismantled in the laboratory to count *P. downsi* larvae, pupae and empty puparia. Parasite load was defined as the total number of *P. downsi* larvae, pupae and empty puparia in a nest.

**Figure 1:**
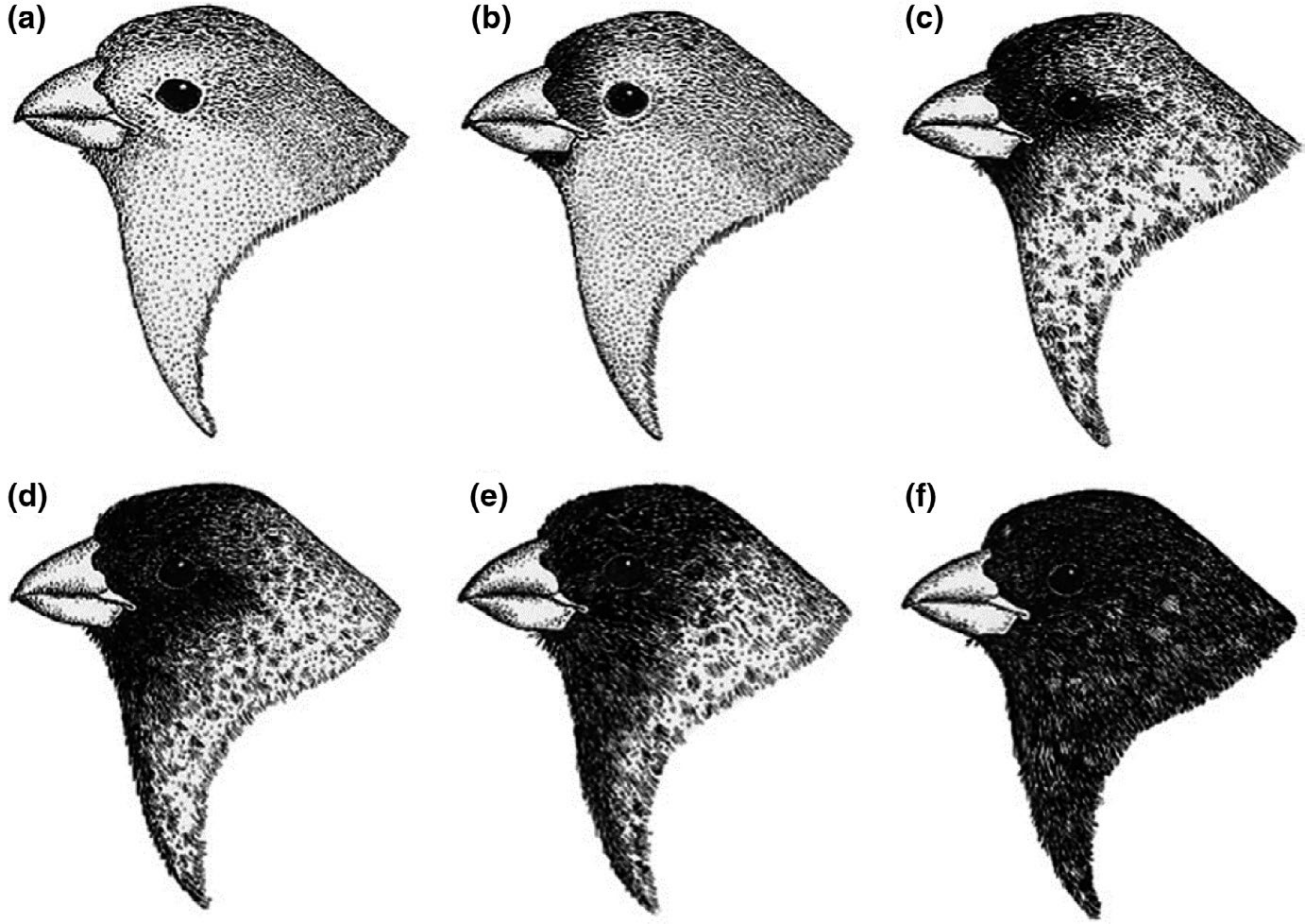
Head, breast and nape plumage of a male small tree finch (*Camarhynchus parvulus*) in yearly intervals, from 0 to ≥5 years of age. Image from Kleindorfer (2007)

The male of each nest was assigned to one of the age‐dependent head colour categories defined by Kleindorfer (2007): (a) wholly brown plumage, (b) small patch of black plumage near the beak, (c) black plumage reaching the eye, (d) black plumage extending beyond the eye, (e) black plumage extending to throat but not nape and (f) wholly black head, throat and nape (Figure 1). As the pattern changes due to annual moults, intervals between categories were considered to be one year.

Nests in close spatial and temporal proximity to a previously monitored nest were considered renesting attempts by the same breeding pair. In such cases, and with pairs where the male was ringed, only one randomly selected nest per breeding season was included in the data set. Data from 2017 were excluded from analysis because we specifically wanted to test the influence of parasitism on breeding success. In 2017, the number of fledged chicks per nest was significantly higher than in 2012–2016 (Mann–Whitney *U* = 4,016, *p* < .001) and *P. downsi* abundance significantly lower than in the other years (Cimadom et al., 2019). This resulted in a sample size of 238 nests for analysis of breeding success (see Table 1).

**Table 1:** Number of analysed small tree finch nests per male age in the years 2012, 2014, 2015, 2016 and total. Number of analysed nests with nestlings ≥6 days in parentheses

Year	Age category						Total
	a	b	c	d	e	f	
2012	4 (1)	6 (1)	2 (1)	11 (2)	12 (7)	13 (3)	48 (15)
2014	10 (2)	6 (1)	9 (5)	18 (7)	13 (8)	23 (10)	79 (33)
2015	17 (6)	7 (4)	4 (1)	18 (12)	14 (4)	6 (3)	66 (30)
2016	13 (3)	4 (1)	6 (3)	8 (2)	12 (5)	2 (0)	45 (14)
Total	44 (12)	23 (7)	21 (10)	55 (23)	51 (24)	44 (16)	238 (92)

### 2.3 Permethrin treatment

To assess differences between young and old males in the absence of *P. downsi* infestation, 45 additional nests were injected with 10 ml of a 1% solution of the insecticide permethrin (Permectrin™ II) in the years 2015 and 2016. Injection took place in a window of three days before or after hatching and was performed only once. The insecticide was injected into the bottom layer of the nest via a pole‐mounted 5‐ml syringe. Due to the location of the nests on thin branches in heights of 5 m or more, chicks or eggs could not be removed from the nest for the duration of the treatment, but great care was taken to avoid chicks and eggs coming into direct contact with the permethrin solution. Five nests were abandoned within one day after treatment and excluded from further analysis, resulting in a total sample size of 40 treated nests. Untreated nests with chicks in the years 2015 and 2016 were used as a control group (*n* = 78, see Table 2). Permethrin treatment was successful and led to a significant reduction in parasite load (mean ± standard error, permethrin: 9.82 ± 2.83; untreated: 37.4 ± 3.0) and to a significantly higher breeding success (mean number of fledglings per nest ± standard error, permethrin: 1 ± 0.17; untreated: 0.15 ± 0.06) (see also Cimadom et al., 2019). Nests were not strictly randomly assigned to the permethrin treatment but rather according to accessibility. This is due to the fact that permethrin treatment could only be carried out in nests that were A) found before hatching of the chicks and B) within reach of the pole‐mounted syringe. This approach resulted in treated nests being, on average, lower than untreated nests (nest height mean ± *SD*: treated nests: 6.4 ± 1.0 m, untreated nests: 7.2 ± 1.4 m; Welch two‐sample *t* test, *t* = −3.92, *df* = 106.78, *p*‐value < .001). However, there was no difference in nest height between the two age groups (see results) and the distribution of nests of young and old males was the same in both treatment groups (χ^2^ = 0.15, *df* = 1, *p*‐value = .7).

**Table 2:** Number of small tree finch nests (nests with chicks) treated with permethrin and untreated nests per age category in the years 2015 and 2016, and total

Year	Treatment	Age category						Total
		a	b	c	d	e	f	
2015	Permethrin	6	4	6	4	3	6	29
	Untreated	11	6	4	16	9	6	52
2016	Permethrin	5	0	1	1	3	1	11
	Untreated	5	3	4	4	9	1	26

### 2.4 Foraging behaviour

To assess whether young and old males differ in their foraging techniques and success, the foraging behaviour of 154 small tree finch males was observed during the breeding season from January–April 2014 (for details see Filek, Cimadom, Schulze, Jäger, & Tebbich, 2018). The first time that an observed bird was seen foraging is referred to as “first foraging observation.” For each first foraging observation, foraging substrate (dead leaf still attached to branch, leaf, moss, twig, bark, *Scalesia* seed stems, herb and soil), prey type (animal or plant) and foraging success (yes or no) were recorded. Foraging success was defined as successful intake of an animal prey item. Observations were conducted along trails to minimize the possibility of repeated observations of the same bird individual during a day. Nevertheless, it was impossible to exclude the possibility of individuals entering the data set more than once, except in the case of banded birds.

### 2.5 Statistical analysis

#### 2.5.1 Age categorization

Using all six male age categories in analysis was not possible as some age categories raised zero chicks in some years. Sample size was not large enough in each of the six categories, particularly for the analysis of the impact of parasitism on the breeding success. Thus, the six categories were collapsed into two age classes. Using two age classes only allows for rather conservative estimates of age‐dependent effects, as the shape of the relation between age and different life history traits can be of different forms (e.g. no age‐dependent effect, thresholds at specific ages and steady increase which flattens out or even decreases late in life as sign of senescence).

Kleindorfer (2007) split males into two groups depending on their head colouration. Categories a–d were considered “brown,” and e–f were defined as “black.” We used the same categorization and terminology for the analysis that focused on the comparison of the data set with Kleindorfer (2007). As we were interested in the effect of (breeding) experience on the reproductive success, we split the age groups differently. Age‐dependent effects are often strongest in young and first‐time breeders compared to older more experienced breeders (Martin, 1995). Thus, age categories a–b were considered inexperienced (with an experience of a maximum of two breeding seasons), while categories c–f were considered experienced (with an experience of more than two breeding seasons). From here on, “young” and “old” will be used to refer to age categories a–b and c–f, respectively (Figure 1).

#### 2.5.2 Changes over time

We used chi‐square tests to compare the frequencies of nesting outcomes in 2012, 2014, 2015 and 2016 with mean values from the years 2000, 2001, 2002 and 2004 reported by Kleindorfer (2007).

A two‐sample *t* test was used to compare clutch size between nests of brown and black males in 2012–2016. Clutch size of brown and black males from 2012, 2014, 2015 and 2016 was compared with the mean value from the years 2000, 2001, 2002 and 2004 reported by Kleindorfer (2007) using a one‐sample *t* test. Nest height from 2012 to 2016 was compared between nests of brown and black males using a two‐sample *t* test. No statistical comparison with the data of Kleindorfer (2007) was made for nest height, as this variable was estimated and we could not account for interobserver reliability.

#### 2.5.3 Breeding success

Breeding success was defined as the number of fledglings per nest. Because of the high number of failed nests, a zero‐inflated generalized linear model (GLM, Poisson with logit link) was calculated to analyse the influence of year (2012/2014/2015/2016), male age (young/old) and nest height on breeding success for untreated nests.

To analyse the effect of male age on breeding success in the presence and absence of *P. downsi* parasitism, we calculated a zero‐inflated GLM with male age (young/old), treatment (permethrin/untreated) and the interaction between male age and treatment as predictors.

#### 2.5.4 Parasite load and predation

The influence of year (2012/2014/2015/2016), male age (young/old), nest height and the interaction between male age and chick age at termination of breeding activity on parasite load (number of *P. downsi* per nest) was investigated with a GLM with a quasi‐Poisson error structure because of overdispersion. Although the impact of *P. downsi* decreases with brood size (Fessl et al., 2006), the number of *P. downsi* per chick was not used as a response variable because clutch size did not differ significantly between male age groups (Mann–Whitney *U* = 7,539.5, *p* = .58). A binomial GLM was calculated to assess the impact of year (2012/2014/2015/2016), male age (young/old) and nest height on predation.

#### 2.5.5 Foraging behaviour

To test whether foraging success (yes/no) of invertebrate prey differed between young and old males, we used a GLM (binomial with logit link). Dead *S. pedunculata* leaves still attached to the branch, which often contain energy‐rich prey such as spiders, beetles, caterpillars and orthopterans, were the most frequently used substrate (Filek et al., 2018). Accordingly, we used a GLM (binomial with logit link) to analyse whether foraging in dead leaves (yes/no) was more common in young or old males.

All statistical analyses were conducted with R, version 3.5.1 (R Core Team, 2018), within RStudio, version 1.1.423 (RStudio Team, 2018). GLMs were calculated using “zeroinfl” function from the “pscl” package (zero‐inflated GLMs) and “glm” function from the “stats” package (quasi‐Poisson and binomial GLMs).

## 3 RESULTS

### 3.1 Changes over time

**Figure 2:**
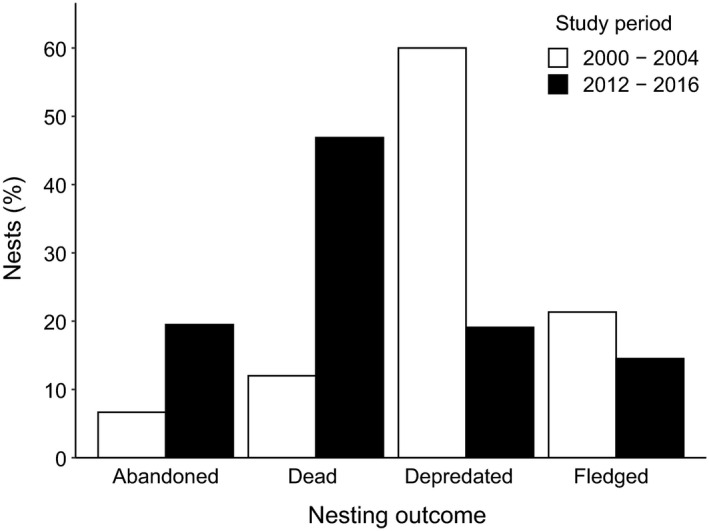
Nesting outcomes (%) of small tree finch nests in the years 2000–2004 (*n* = 75) and 2012–2016 (*n* = 266). Data from 2000 to 2004 from Kleindorfer (2007)

In the years 2012–2016, significantly more nests were abandoned (χ^2^ test, *n*_2012–2016_ = 266, *n*_2000–2004_ = 75, χ^2^ = 4.66, *df* = 1, *p* = .031, Figure 2) and total brood loss with dead chicks still in the nest occurred significantly more often than in the years 2000–2004 (χ^2^ test, *n*_2012–2016_ = 266, *n*_2000–2004_ = 75, χ ^2^ = 22.35, *df* = 1, *p* < .01, Figure 2). Predation was significantly less common in 2012–2016 than in 2000–2004 (χ^2^ test, *n*_2012–2016_ = 266, *n*_2000–2004_ = 75, χ^2^ = 52.38, *df* = 1, *p* < .01, Figure 2). The number of nests with fledglings did not differ significantly between the years 2000–2004 and 2012–2016 (χ^2^ test, *n*_2012–2016_ = 266, *n*_2000–2004_ = 75, χ^2^ = 2.46, *df* = 1, *p* = .116, Figure 2).

**Figure 3:**
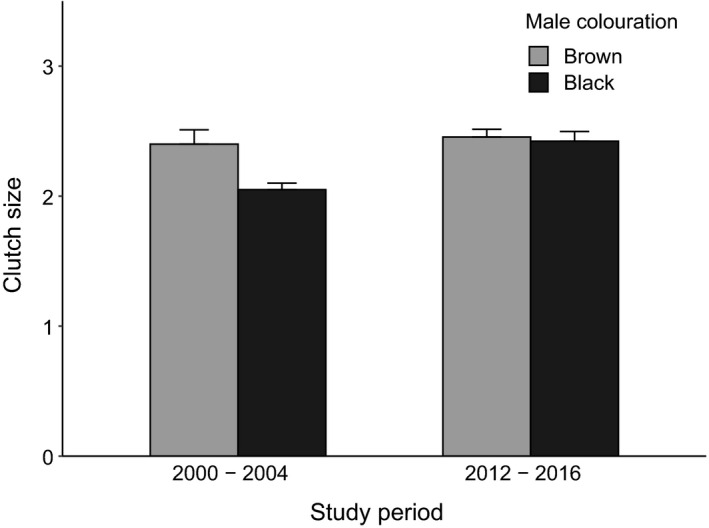
Clutch size (mean ± standard error) of nests of brown (age categories a–d) and black (age categories e–f) males in the years 2000–2004 (*n* = 41) and 2012–2016 (*n* = 240). Data from 2000 to 2004 from Kleindorfer (2007)

**Figure 4:**
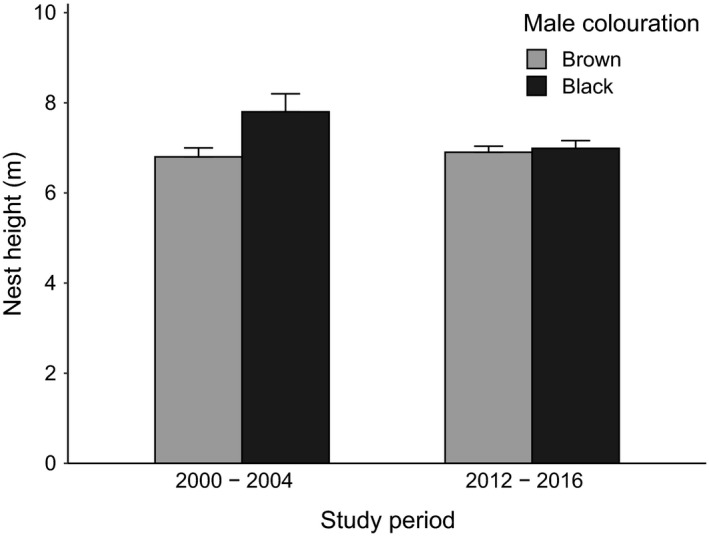
Nest height (mean ± standard error) of brown (age categories a–d) and black (age categories e–f) males in the years 2000–2004 (*n* = 41) and 2012–2016 (*n* = 238). Data from 2000 to 2004 from Kleindorfer (2007)

Clutch size did not differ significantly in nests of brown males between 2000–2004 and 2012–2016 (one‐sample *t* test, *n*_2000–2004_ = 41, *n*_2012–2016_ = 67, *t* = 0.92, *df* = 142, *p* = .359, Figure 3). Nests of black males contained significantly larger clutches in 2012–2016 compared to 2000–2004 (one‐sample *t* test, *n*_2000–2004_ = 41, *n*_2012–2016_ = 171, *t* = 5, *df* = 96, *p* < .001, Figure 3). There was no difference in clutch size between nests of brown and black males in the years 2012–2016 (two‐sample *t* test, *n*_2012–2016_ = 240, *t* = 0.33, *df* = 201.57, *p* = .738, Figure 3). Nest height did not differ between brown and black males in the years 2012–2016 (two‐sample *t* test, *n*_2012–2016_ = 238, *t* = −0.4, *df* = 195.38, *p* = .69, Figure 4).

### 3.2 Breeding success

**Figure 5:**
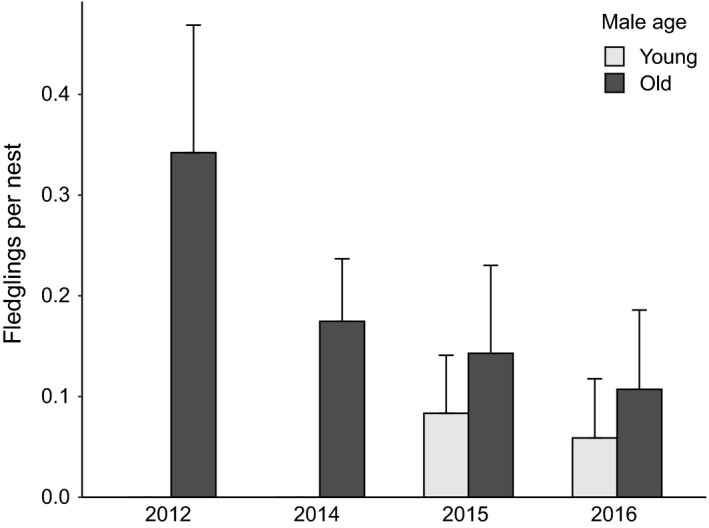
Number of fledglings per nest (mean ± standard error) of young (age categories a–b) and old (age categories c–f) small tree finch males in 2012, 2014, 2015 and 2016 (*n* = 238, see Table 1)

**Figure 6:**
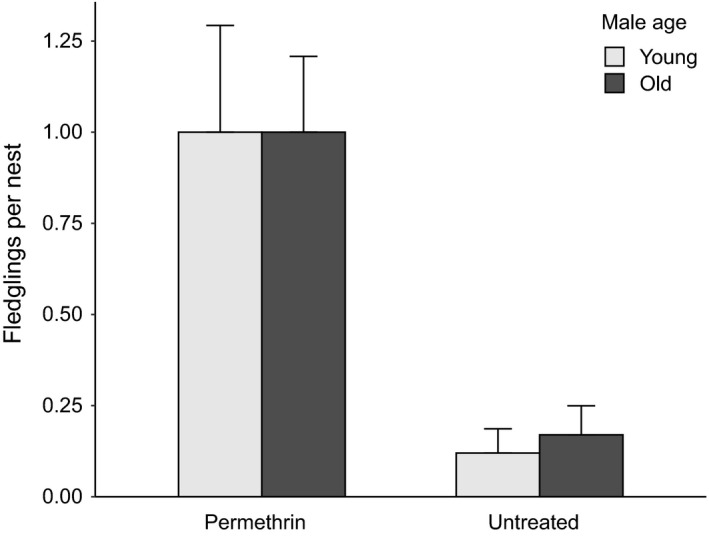
Number of fledglings (mean ± standard error) in nests of young (age categories a–b) and old (age categories c–f) small tree finch males depending on treatment with the insecticide permethrin in the years 2015–2016 (*n* = 118, see Table 2)

The zero‐inflated GLM with number of fledglings as a response variable showed a significant influence of male age, with higher numbers of chicks fledging from the nests of older males (*n* = 238, Table 3, Figure 5). In the permethrin experiment, the zero‐inflated GLM with number of fledglings as a response variable showed a significant influence of the interaction between male age and treatment (Table 4). Nests of old males produced more fledglings than nests of young males, but only under high parasite pressure (untreated nests). The number of fledglings did not differ between age groups among treated nests. However, there was a strong difference between age groups among untreated nests. In these nests, 41.5% more chicks fledged from nests of old males compared to those of young males (Figure 6).

**Table 3:** Results of zero‐inflated GLM testing for effects of male age on the number of fledglings per nest (*n* = 238, see Table 1)

Response Variable: Number of Fledglings				
Predictors	Estimate	*SE*	*z*	*p*‐Value
Count model coefficients (Poisson with log link)				
Intercept	−2.42	1.39	−1.75	.08
Male age (young/old)	3.36	0.66	5.11	<.001
Year 2014	−0.81	0.68	−1.18	.24
Year 2015	0.41	0.6	0.69	.49
Year 2016	−0.20	0.8	−0.25	.81
Nest height	−0.10	019	−0.56	.58
Zero‐inflation model coefficients (binomial with logit link)				
Intercept	−13.89	594.24	−0.02	.98
Male age (young/old)	14.40	594.24	0.02	.98
Year 2014	−0.30	0.92	−0.33	.74
Year 2015	1.18	0.83	1.41	.16
Year 2016	0.86	1.04	0.82	.41
Nest height	0.10	0.23	0.41	.68

Abbreviation: *SE*, standard error.

**Table 4:** Results of zero‐inflated GLM testing for effects of male age, treatment and the interaction term male age × treatment on the number of fledglings in small tree finch nests. (*n* = 118, see Table 2)

Response Variable: Number of Fledglings				
Predictors	Estimate	*SE*	*z*	*p*‐Value
Count model coefficients (Poisson with log link)				
Intercept	0.35	0.35	1.01	.31
Male age (young/old)	−0.09	0.45	−0.21	.84
Treatment (permethrin/untreated)	−2.47	0.68	−3.66	<.001
Male age × treatment	2.49	0.87	2.87	.004
Zero‐inflation model coefficients (binomial with logit link)				
Intercept	−0.86	0.97	−0.89	.38
Male age (young/old)	−0.35	1.37	−0.26	.80
Treatment (permethrin/untreated)	−6.75	101.90	−0.70	.95
Male age × treatment	9.88	101.90	0.10	.92

Abbreviation: *SE*, standard error.

### 3.3 Parasite load and predation

Male age, year, nest height, the interaction between male age and year and the interaction between male age and age of chicks at termination of breeding activity did not predict *P. downsi* parasite load per nest. Age of chicks at termination of breeding activity was positively correlated with *P. downsi* parasite load (Table 5). Also, nest predation was not influenced by male age, year or the interaction term male age × year (Table 6).

**Table 5:** Results of quasi‐Poisson GLM testing for effects of male age, chick age at termination of breeding activity, year and the interaction term male age × year on the number of *Philornis downsi* per nest (*n* = 209)

Response Variable: Number of *Philornis downsi* larvae and pupae per nest				
Predictors	Estimate	*SE*	t	*p*‐Value
Intercept	2.92	0.28	10.41	<.001
Male age (young/old)	0.03	0.23	0.15	.88
Chick age	0.08	0.02	3.32	.001
Year (2014)	−0.22	0.13	−1.7	.09
Year (2015)	0.09	0.13	0.72	.47
Year (2016)	−0.05	0.16	−0.35	.73
Nest height	−0.01	0.03	−0.16	.87
Male age × chick age	0.02	0.03	0.63	.53

Abbreviation: *SE*, standard error.

**Table 6:** Results of binomial GLM testing for effects of male age, year and the interaction term male age × year on nest predation (*n* = 238, see Table 1)

Response Variable: Nest predation				
Predictors	Estimate	*SE*	*z*	*p*‐Value
Intercept	−2.91	1.14	−2.54	.011
Male age (young/old)	0.26	0.55	0.48	.63
Year (2014)	0.78	0.69	1.12	.26
Year (2015)	0.90	0.71	1.27	.2
Year (2016)	−15.81	971.13	−0.02	.99
Nest height	−0.00	0.14	−0.02	.99

Abbreviation: *SE*, standard error.

### 3.4 Foraging behaviour

**Figure 7:**
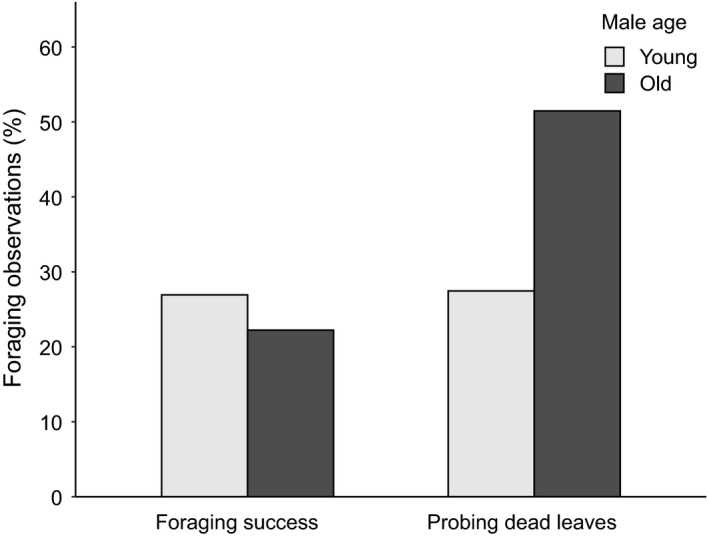
Percentage of successful first foraging observations and “probing dead leaves” foraging observations of young (age categories a–b) and old (age categories c–f) males

Foraging success did not differ between young and old males (GLM, estimate = −0.254, *SE* = 0.525, *z* = −0.484, *p* = .628, Figure 7). However, old males were more often observed searching for food in dead leaves still attached to the branch than young males (GLM, estimate = 1.03, *SE* = 0.371, *z* = 2.78, *p* = .005, Figure 7).

## 4 DISCUSSION

We found that old males had a significantly higher breeding success than young, inexperienced males, but only in nests containing high numbers of parasitic *P. downsi* larvae. In comparison with the studies by Kleindorfer (2007) and Dudaniec, Fessl, and Kleindorfer (2007), two main reasons for breeding failure have changed: predation and parasitism. In 2012–2016, the level of predation was significantly lower than in 2000–2004. Parasitism by *P. downsi* showed an opposite trend and was significantly higher in 2012–2016. We do not know the reason for the reduction in predation and, thus, can only speculate. Two native predators of small tree finch nests, the barn owl (*Tyto alba*) and short‐eared owl (*Asio flammeus*), often fall victim to the Santa Cruz Highway, which bisects the study area (pers. observation). Their populations may have decreased since 2000–2004. Alternatively, due to higher *P. downsi* parasitism, nestlings die younger (Kleindorfer & Dudaniec, 2016) and thus do not reach the age when loud begging calls are emitted (from day six on). Begging calls make nests more conspicuous for predators (Ibáñez‐Álamo, Arco, & Soler, 2012). Furthermore, predation did not differ significantly between young and old males. In the study by Kleindorfer (2007), reduced predation of nests of black males was explained by increased nest height and concealment. In our study, nest concealment was not measured, but an analysis of nest height revealed no significant differences between brown and black males in the years 2012–2016. It is possible that the more uniform nesting height found in 2012–2016 can be explained by more uniform canopy height caused by synchronized collapse in wet years. *Scalesia pedunculata* is known to be subject to this phenomenon (Kastdalen, 1982). Repeated catastrophes such as El Niño events are hypothesized to lead to the forest becoming progressively more uniform (Lawesson, 1988). Despite the decline in predation, overall fledging success was not significantly higher in 2012–2016 than in 2000–2004. In the absence of strong predation pressure, there was a significant increase in the abandonment of eggs and total brood loss with dead chicks still in the nest. Therefore, it stands to reason that the influence of other detrimental factors has increased since 2004.

The most obvious candidate is the parasitic fly *Philornis downsi*. Cimadom et al. (2014) found *P. downsi* to be responsible for 56% of dead small tree finch nestlings in 2010 and 2012. Long‐term data showed that the abundance of *P. downsi* larvae in nests of several species of Darwin's finches has increased significantly across multiple islands between the years 2000 and 2014 (Kleindorfer & Dudaniec, 2016). However, we found no difference in parasite load between nests of young and old males, and therefore, the lower breeding success of young males cannot be attributed to higher susceptibility to infestation by *P. downsi*. Furthermore, the difference in breeding success between young and old males disappeared in parasite‐reduced nests. This suggests that old males are better at compensating for parasites. One possible trait that could allow old males to compensate is an increased feeding rate. In several songbird species, parents can reduce the effect of parasitism on nestlings by increasing feeding rates (Bennett & Whitworth, 1991; Christe, Richner, & Oppliger, 1996; Fessl et al., 2006) but this ability was not observed in small tree finches (Heyer et al., 2020) and two species of Darwin's ground finches (Knutie et al., 2016; O’Connor, Robertson, & Kleindorfer, 2014).

We did not measure parental food provisioning rates of small tree finches in the years 2012–2016. However, we measured foraging success, which is the presumed pre‐condition for the ability to provide more or better quality food to chicks. In birds, foraging success generally improves with age (Wunderle, 1991) due to use of better foraging sites (American redstarts, *Setophaga ruticilla* (Ficken & Ficken, 1967)), more efficient search methods (American robins, *Turdus migratorius* (Gochfeld & Burger, 1984); bananaquits, *Coereba flaveola* (Wunderle & Lodge, 1988)), more complex foraging techniques (reed warblers, *Acrocephalus scirpaceus* (Davies & Green, 1976); northern mockingbirds, *Mimus polyglottos* (Breitwisch, Lee, & Diaz, 1987)), selection of more suitable food items (red‐winged blackbirds, *Agelaius phoeniceus* (Alcock, 1973)) and more efficient food handling (reed warblers (Davies & Green, 1976); Eurasian nuthatches, *Sitta europea* (Enoksson, 1988)). While we found no difference in foraging efficiency between young and old males, we observed that old males were more likely to probe dead *Scalesia pedunculata* leaves still attached to the branch. Dead leaves often contain high quantities of prey items with high energy content, such as caterpillars and orthopterans, which are important for chick rearing (Remsen & Parker, 1984; Sutherland, Newton, & Green, 2004). However, this extractive foraging behaviour involves a complex sequence of a sensory–motor pattern similarly to string‐pulling (Seibt & Wickler, 2006) and could be a motor pattern that improves with age due to learning. Furthermore, it is possible that young birds need time to learn about the valuable prey items hidden in dead leaves.

However, besides age‐dependent differences in experience, Forslund and Pärt (1995) review two alternative theories for increasing breeding success with increasing age: optimization of reproductive effort and progressive appearance or disappearance of phenotypes. Due to the fact that the likelihood of surviving the next year decreases with age, older birds have a stronger incentive to invest more resources into breeding, thus optimizing their reproductive effort. This hypothesis is not well supported by our data. If older males are investing more in reproduction, breeding success should be also age‐dependent when parasites are removed.

Progressive appearance or disappearance of phenotypes occurs when individuals of different phenotypic quality differ in age of first reproduction or survival probability, and therefore, the proportion of individuals of different phenotypic quality will change between age classes. If birds with higher reproductive success have a higher likelihood of surviving any given year, the proportion of good reproducers and thus average reproductive success will be higher in older than younger age classes. To test this hypothesis, more data on survival probability and breeding performance of individual males would be needed.

Differences in breeding success between young and old males may also be attributed to female strategies which allow them to adjust to male quality. Females could respond to high‐quality mates by investing in higher‐quality eggs/offspring—for example by adjusting yolk testosterone levels (Gwinner, Yohannes, & Schwabl, 2013; Navara, Hill, & Mendonça, 2006), which could also potentially lead to higher *P. downsi* tolerance (regardless of differences in foraging ability). Kleindorfer (2007) found that females paired with brown males had significantly larger clutch sizes, but during our study period, the difference in clutch size had disappeared. It is possible that, due to increased parasite pressure, females adjust clutch size to the largest number of nestlings they can, on average, provide with sufficient food (Lack, 1954). This would be done independently of the age of the male partner, in order to dilute the impact of *P. downsi* on each individual chick.

In conclusion, nests of old males had a significantly higher number of fledglings than nests of young males. However, the effect of male age is also dependent on the level of parasitism, which varies between years. It seems that differences in breeding success between young and old males are exacerbated under adverse conditions such as strong pressure from predators and parasites. Old males are likely not just better in food provisioning, but their experience allows them to better cope with adverse conditions in general.

## CONFLICT OF INTEREST

The authors declare that no competing interests exist.
